# Crystal structure, Hirshfeld analysis and mol­ecular docking with the vascular endothelial growth factor receptor-2 of (3*Z*)-5-fluoro-3-(hy­droxy­imino)­indolin-2-one

**DOI:** 10.1107/S2056989017008301

**Published:** 2017-06-07

**Authors:** Bianca Barreto Martins, Leandro Bresolin, Renan Lira de Farias, Adriano Bof de Oliveira, Vanessa Carratu Gervini

**Affiliations:** a Universidade Federal do Rio Grande (FURG), Escola de Química e Alimentos, Rio Grande, Brazil; b Universidade Estadual Paulista (UNESP), Instituto de Química, Araraquara, Brazil; c Universidade Federal de Sergipe (UFS), Departamento de Química, São Cristóvão, Brazil

**Keywords:** crystal structure, Hirshfeld surface analysis, isatin derivative–VEGFR-2 *in silico* evaluation

## Abstract

The reaction between 5-fluoro­isatin and hydroxyl­amine hydro­chloride in acidic ethanol yields the title compound, C_8_H_5_FN_2_O_2_, whose mol­ecular structure matches the asymmetric unit and is nearly planar with an r.m.s. deviation for the mean plane through all non-H atoms of 0.0363 Å. In the crystal, the mol­ecules are linked by N—H⋯N, N—H⋯O and O—H⋯O hydrogen-bonding inter­actions into a two-dimensional network along the (100) plane, forming rings with *R_2_^2^*(8) and *R_1_^2^*(5) graph-set motifs. The crystal packing also features weak π–π inter­actions along the [100] direction [centroid-to-centroid distance 3.9860 (5) Å]. Additionally, the Hirshfeld surface analysis indicates that the major contributions for the crystal structure are the O⋯H (28.50%) and H⋯F (16.40%) inter­actions. An *in silico* evaluation of the title compound with the vascular endothelial growth factor receptor-2 (VEGFR-2) was carried out. The title compound and the selected biological target VEGFR-2 show the N—H⋯O(*GLU94*), (*CYS96*)N—H⋯O(isatine) and (*PHE95*)N—H⋯O(isatine) inter­molecular inter­actions, which suggests a solid theoretical structure–activity relationship.

## Abstract 2

The mol­ecular structure of 5-fluoro­isatin-3-oxime matches the asymmetric unit and is nearly planar. The mol­ecules are linked into a two-dimensional hydrogen-bonded network parallel to the (100) plane and the Hirshfeld surface analysis indicates that the major contributions for the crystal structure cohesion are the O⋯H (28.5%) and H⋯F (16.4%) inter­actions. An *in silico* evaluation of the title compound with the VEGFR-2 kinase was carried out and suggests a solid theoretical structure–activity relationship.

## Chemical context   

The chemistry of isatin is already well documented due to its wide range of applications, especially in organic synthetic chemistry and medicinal chemistry. The first reports on the synthesis of isatin and isatin-based derivatives can be traced back to the first half of the 19th century (Erdmann, 1841*a* ▸,*b* ▸; Laurent, 1841 ▸) and almost one hundred years after those publications, the review ‘The Chemistry of Isatin’ showed the versatility of this mol­ecular fragment (Sumpter, 1944 ▸). Two recent examples of this are the synthesis of 1-[(2-methyl­benzimidazol-1-yl) meth­yl]-2-oxo-indolin-3-yl­idene]amino]­thio­urea, an *in vitro* and *in silico* Chikungunya virus inhibitor (Mishra *et al.*, 2016 ▸) and 5-chloro­isatin-4-methyl­thio­semi­carbazone, an inter­mediate in the HIV-1 (human immuno­deficiency virus type 1) RT (reverse transcriptase) inhibitor (Meleddu *et al.*, 2017 ▸). For these reasons, the crystal structure determination of isatin-based mol­ecules is an intensive research field and one of our major research aims. Herein, the structure, the Hirshfeld surface analysis and the mol­ecular docking with the vascular endothelial growth factor receptor-2 (VEGFR-2) of the 5-fluoro­isatin-3-oxime are reported.
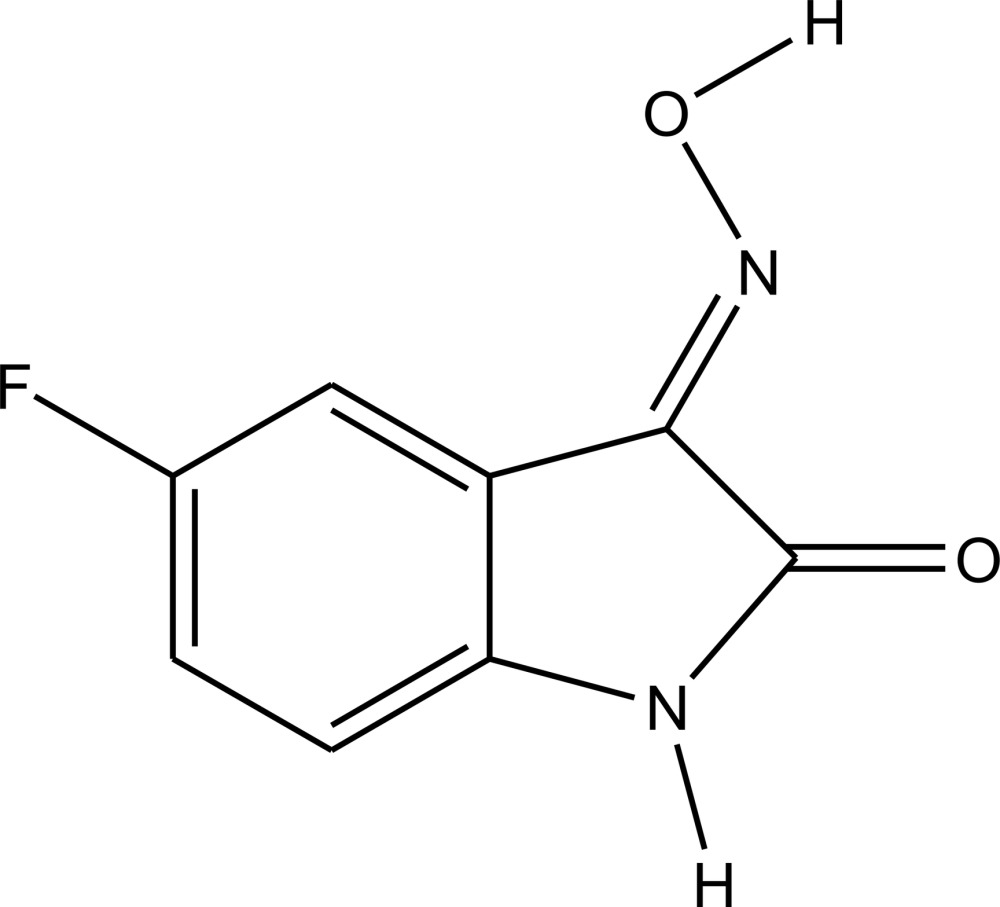


## Structural commentary   

**Figure 1:**
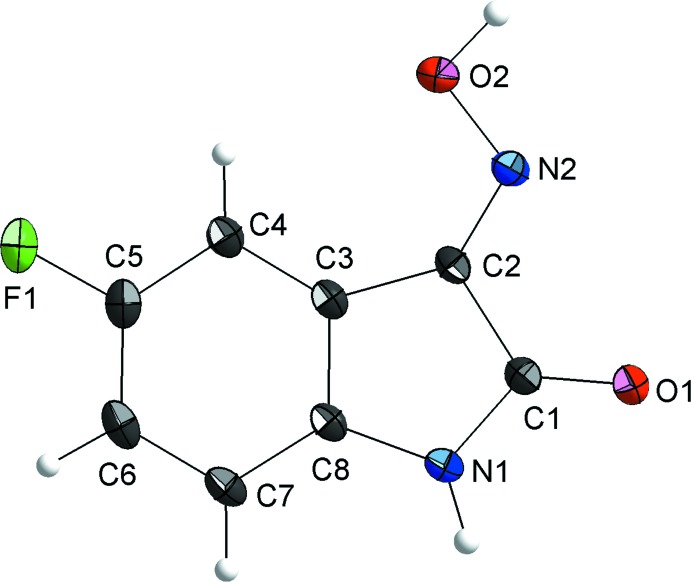
The mol­ecular structure of the title compound with displacement ellipsoids drawn at the 40% probability level.

The mol­ecular structure of the title compound (Fig. 1 ▸) matches the asymmetric unit and it is nearly planar with an r.m.s. deviation from the mean plane of the non–H atoms of 0.0363 Å [from −0.0806 (9) Å for atom O2 to 0.0575 (11) Å for atom C2]. The C1—C2—N2—O2 and C3—C2—N2—O2 torsion angles are −174.24 (10) and −0.5 (2)°, respectively.

## Supra­molecular features and Hirshfeld surface analysis   

**Figure 2:**
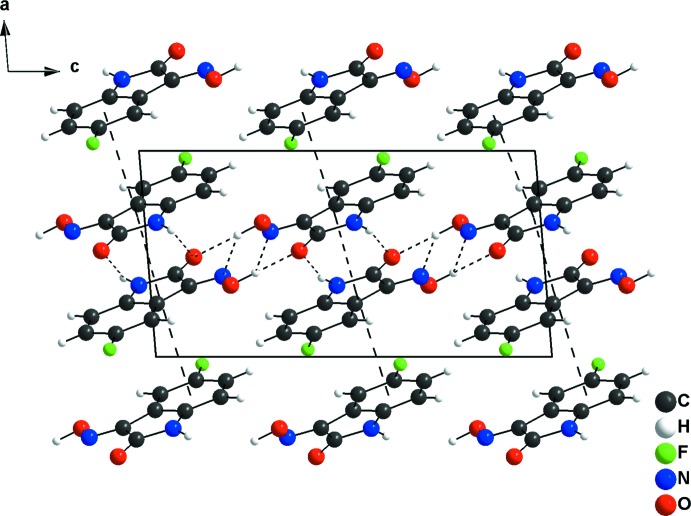
Crystal structure of the title compound viewed along the [010] direction. The H⋯O and H⋯N inter­actions in the crystal packing are shown as dashed lines and connect the mol­ecules into a two-dimensional H-bonded network along the (100) plane. The *Cg*⋯*Cg* packing along the [100] direction is also shown as dashed lines.

In the crystal, the mol­ecules are connected by centrosymmetric pairs of N1—H4⋯O1^*i*^ [symmetry code: (i) −*x* + 1, −*y* + 2, −*z* + 1] inter­molecular inter­actions into dimers with graph-set motif *R_2_^2^*(8) (Table 1 ▸). In addition, a remarkable feature consists in an asymmetric bifurcated hydrogen bond with graph-set motif *R_1_^2^*(5) involving the H5 atom of the oxime group and the O1^ii^ and N2^ii^ atoms of a neighboring mol­ecule [symmetry code: (ii) −*x* + 1, *y* − 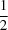, −*z* + 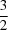]. These two hydrogen bonds, which form rings with motifs *R_2_^2^*(8) and *R_1_^2^*(5), connect the mol­ecules into a two-dimensional, tape-like network parallel to the (100) plane. Finally, the mol­ecules are stacked along the [100] direction by weak π–π inter­actions (Fig. 2 ▸) between the benzene and the indolic five-membered rings. The centroid-to-centroid distance is 3.9860 (5) Å).

**Figure 3:**
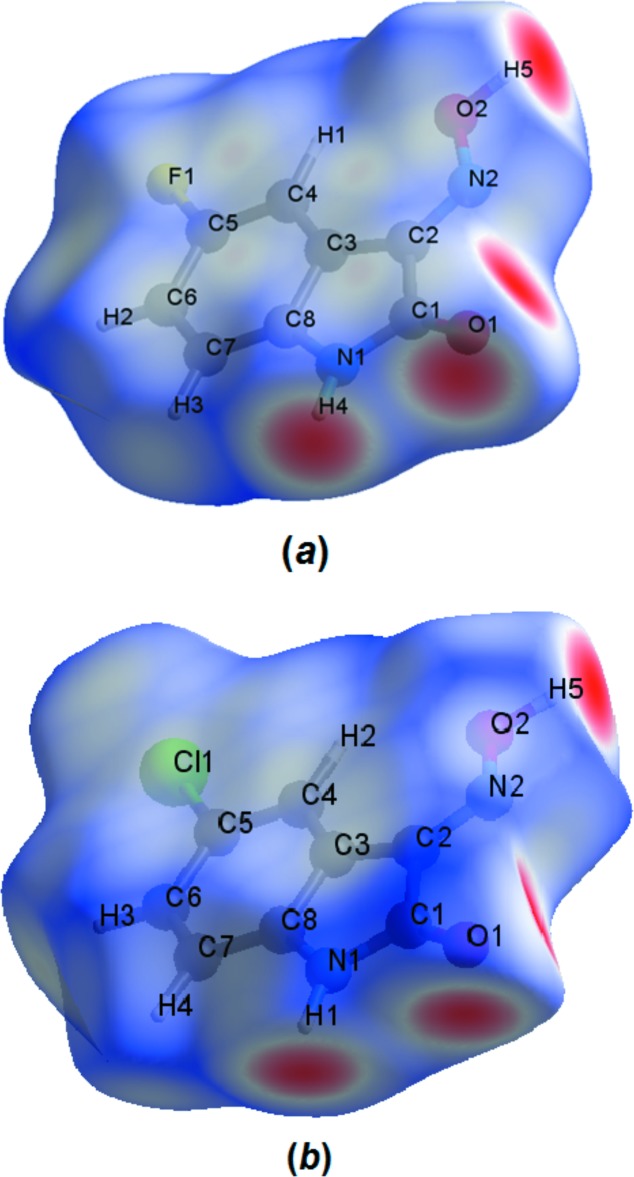
The Hirshfeld surface graphical representation (*d_norm_*) for the asymmetric unit of (*a*) the title compound, 5-fluoro­isatin-3-oxime, and (*b*) the comparison compound, 5-chloro­isatin-3-oxime (Martins *et al.*, 2016 ▸). The surface regions with strongest inter­molecular inter­actions are drawn in magenta colour.

**Figure 4:**
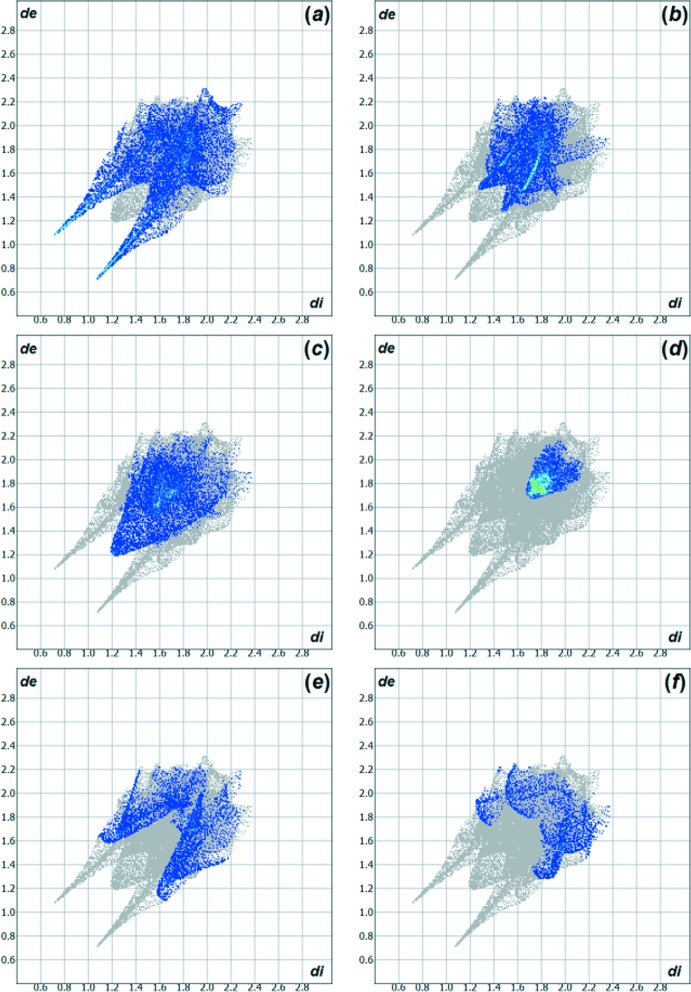
Hirshfeld surface two-dimensional fingerprint plots for the title compound showing the (*a*) H⋯O, (*b*) H⋯F, (*c*) H⋯H, (*d*) C⋯C, (*e*) H⋯N and (*f*) H⋯C contacts in detail (cyan dots). The contributions of the inter­actions to the crystal packing amount to 25.40%, 16.40%, 16.10%, 11.30%, 9.80% and 6.40%, respectively. The *d_e_* (*y* axis) and *d_i_* (*x* axis) values are the closest external and inter­nal distances (values in Å) from given points on the Hirshfeld surface contacts.

The Hirshfeld surface analysis (Hirshfeld, 1977 ▸) of the crystal structure for the title compound was performed. The surface graphical representation, *d_norm_*, with transparency and labelled atoms indicates, in magenta colour, the locations of the strongest inter­molecular contacts, *e.g.* H4, H5 and O1, which are important for the inter­molecular hydrogen bonding (Fig. 3 ▸ *a*). The Hirshfeld analysis suggests that the major contributions for the crystal packing amount to 25.40% for H⋯O, 16.40% for H⋯F and 16.10% for H⋯H inter­actions. Other important inter­molecular contacts for the cohesion of the structure are (values given in %): C⋯C = 11.30, H⋯N = 9.80 and H⋯C = 6.40 (Wolff *et al.*, 2012 ▸; Fig. 4 ▸).

## Comparison with a related structure   

For a comparison with the title compound, 5-fluoro­isatin-3-oxime, the structure of the related compound 5-chloro­isatin-3-oxime (Martins *et al.*, 2016 ▸) was selected. Both structures are nearly planar, build a two-dimensional hydrogen-bonded network parallel to the (100) plane and show the mol­ecules stacked along the [100] direction. The Hirshfeld surface analysis (Hirshfeld, 1977 ▸) for 5-chloro­isatin-3-oxime was carried out and the Hirshfeld surface graphical representation, *d_norm_*, with transparency and labelled atoms indicates, in magenta colour, the locations of the strongest inter­molecular contacts, *e.g.* H1, H5 and O1 (Fig. 3 ▸ *b*). Although the crystal packing (Figs. 2 ▸ and 5 ▸) and the Hirshfeld surface graphical representations (Fig. 3 ▸ *a*,*b*) for the title compound and the 5-chloro­isatin-3-oxime are quite similar, the contributions of the inter­molecular inter­actions to the cohesion of the crystal structures have differences due to the halogen substituents. For example: for 5-chloro­isatin-3-oxime, the H⋯O inter­action amounts to 23.60% and the H⋯Cl inter­action amounts to 18.10%. The contributions to the crystal packing are shown as Hirshfeld surface two-dimensional fingerprint plots with cyan dots. The *d_e_* (*y* axis) and *d_i_* (*x* axis) values are the closest external and inter­nal distances (Å) from given points on the Hirshfeld surface contacts (Figs. 4 ▸ and 6 ▸; Wolff *et al.*, 2012 ▸).

## Mol­ecular docking evaluation   

**Figure 7:**
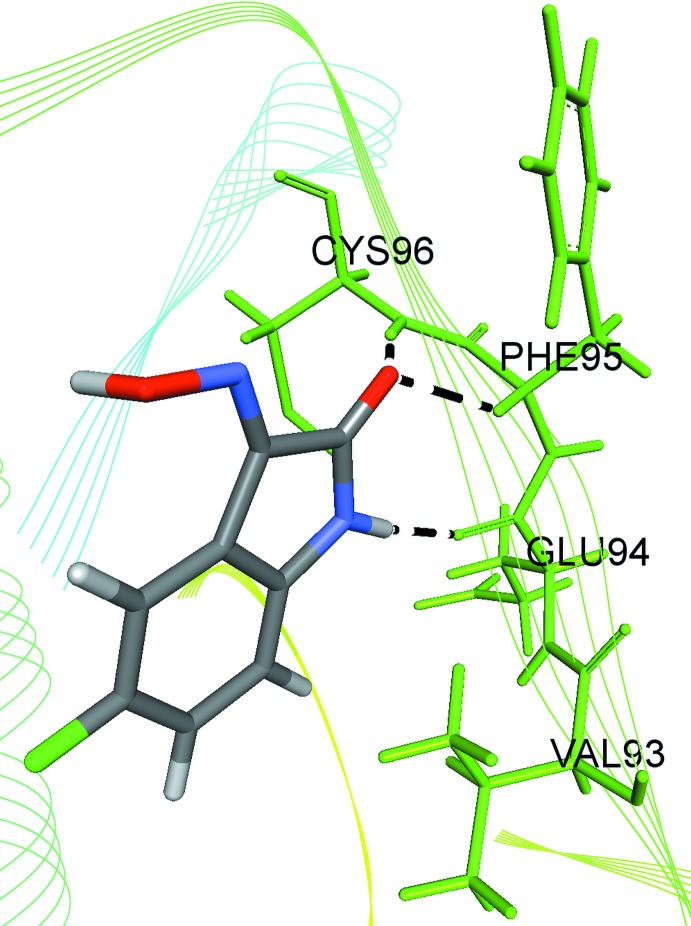
Graphical representation of a lock-and-key model for the inter­molecular inter­actions between the title compound and selected residues of the VEGFR-2. The inter­actions are shown as dashed lines and the structure of the enzyme is simplified for clarity.

For a lock-and-key supra­molecular analysis, a mol­ecular docking evaluation between the title compound and the vascular endothelial growth factor receptor-2 (VEGFR-2) was carried out. Initially, the semi-empirical equilibrium energy of the small mol­ecule was obtained using the PM6 Hamiltonian, but the experimental bond lengths were conserved. The calculated parameters were: heat of formation = −49.353 kJ mol^−1^, gradient normal = 0.90997, HOMO = −9.265 eV, LUMO = −1.337 eV and energy gap = 7.928 eV (Macrae *et al.*, 2008 ▸; Stewart, 2013 ▸, 2016 ▸). The biological target prediction for the title compound was calculated with the *SwissTargetPrediction* webserver based on the bioisosteric similarity to the isatin entity (Gfeller *et al.*, 2013 ▸, 2014 ▸). As result of this screening, the title compound showed a promising theoretical structure–activity relationship to kinase proteins sites: ‘Frequency Target Class’ for kinases amounts to 33% [see the ‘SwissTargetPrediction report (5-fluoro­isatin-3-oxime)’ in the Supporting information]. The protein kinases regulate several critical cellular processes (Wang & Cole, 2014 ▸) and the vascular endothelial growth factor receptor-2 kinase inhibition is becoming an attractive subject for anti­cancer drug research (Gao *et al.*, 2015 ▸). The crystal structure of the vascular endothelial growth factor receptor-2 (VEGFR-2), PDB ID: 3WZD, was downloaded from Protein Data Bank (Okamoto *et al.*, 2015 ▸). Before the calculations, a stereochemical evaluation of the protein structure was carried out using the Ramachandran analysis (Lovell *et al.*, 2003 ▸) and the number of residues in favoured regions for inter­molecular inter­actions was over 98% [see the ‘Number of residues in favoured region (VEGFR-2)’ in the Supporting information]. The docking simulation was performed with the *GOLD 5.5* software (Chen, 2015 ▸) and a grid of 25 Å was centered on the binding site of Levatinib in the VEGFR-2 kinase (Okamoto *et al.*, 2015 ▸). A redocking of the Levatinib compound, an oral multikinase inhibitor that selectively inhibits the vascular endothelial growth factor-2, was used as validation method for the mol­ecular docking protocol (see the ‘Re-docking of the Lenvatinib (kinase inhibitor and FDA approved drug)’ in the Supporting information]. A calculated global free energy of −20.49 kJ mol^−1^ was found for the title compound and the selected biological target VEGFR-2 inter­action and the structure–activity relationship can be assumed by the following observed inter­molecular inter­actions, with the respective hydrogen-bond distances and angles: N—H⋯O(*GLU94*) [H⋯O = 2.03 Å, N—H⋯O = 174°], (*CYS96*)N—H⋯O(isatine) [H⋯O = 1.72 Å, N—H⋯O = 168°] and (*PHE95*)C—H⋯O(isatine) [H⋯O = 2.27 Å, C—H⋯O = 140°] (Fig. 7 ▸). Another significant feature of the structure of the title compound is the oxygen atom of the isatin fragment. The O1 atom is a hydrogen-bond acceptor and bridges two D—H⋯O inter­actions (supra­molecular chemistry, Fig. 2 ▸; Hirshfeld surface, Fig. 3 ▸; mol­ecular docking with the biological target VEGFR-2 kinase, Fig. 7 ▸).

## Synthesis and crystallization   

All starting materials are commercially available and were used without further purification. The synthesis of the title compound was adapted from procedures reported previously (Martins *et al.*, 2016 ▸; O’Sullivan & Sadler, 1956 ▸; Sandmeyer, 1919 ▸; Sumpter, 1944 ▸). A glacial acetic acid catalyzed mixture of 5-fluoro­isatin (3 mmol) and hydroxyl­amine hydro­chloride (3 mmol) in ethanol (50 mL) was stirred and refluxed for 6 h. After cooling and filtering, single crystals suitable for X-ray diffraction were obtained from the ethano­lic solution by solvent evaporation.

## Refinement   

Crystal data, data collection and structure refinement details are summarized in Table 2 ▸. The H4 and H5 atoms were located in a difference Fourier map and freely refined [N1—H4 = 0.91 (2) Å and O2—H5 = 0.99 (3) Å]. The H1, H2 and H3 atoms were positioned with idealized geometry (HFIX command) and refined using a riding model, with C—H = 0.95 Å and *U*_iso_(H) = 1.2*U*_eq_(C).

## Supplementary Material

Crystal structure: contains datablock(s) I. DOI: 10.1107/S2056989017008301/rz5215sup1.cif

Structure factors: contains datablock(s) I. DOI: 10.1107/S2056989017008301/rz5215Isup2.hkl

SwissTargetPrediction report (5-flouroisatin-3-oxime). DOI: 10.1107/S2056989017008301/rz5215sup3.pdf

Number of residues in favoured region (VEGFR-2). DOI: 10.1107/S2056989017008301/rz5215sup4.pdf

Re-docking of the Lenvatinib (kinase inhibitor and FDA approved drug). DOI: 10.1107/S2056989017008301/rz5215sup5.pdf

Supporting information file. DOI: 10.1107/S2056989017008301/rz5215Isup6.cml

CCDC reference: 1554287

Additional supporting information:  crystallographic information; 3D view; checkCIF report

## Figures and Tables

**Figure 5:**
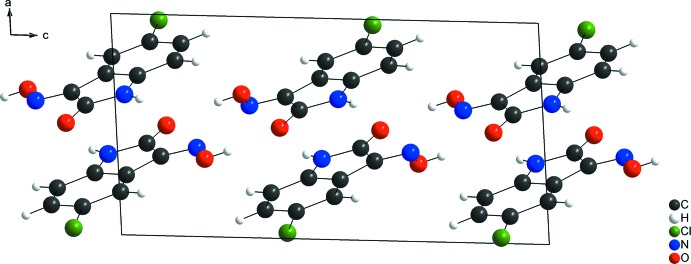
Crystal structure of the comparison compound 5-chloro­isatin-3-oxime (Martins *et al.*, 2016 ▸), viewed along the [010] direction.

**Figure 6:**
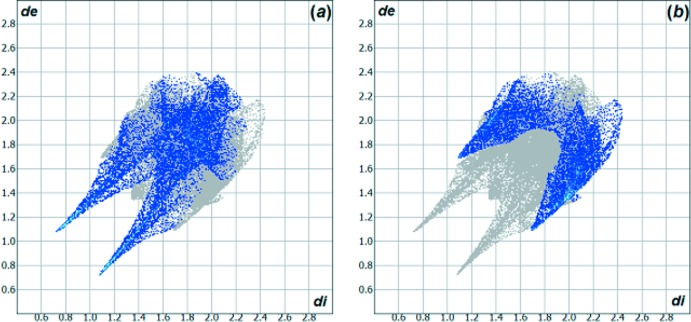
Hirshfeld surface two-dimensional fingerprint plots for the comparison compound 5-chloro­isatin-3-oxime (Martins *et al.*, 2016 ▸) showing the (*a*) H⋯O and (*b*) H⋯Cl contacts in detail (cyan dots). The contributions of the inter­actions to the crystal packing amount to 23.60% and 18.10%. The *d_e_* (*y* axis) and *d_i_* (*x* axis) values are the closest external and inter­nal distances (values in Å) from given points on the Hirshfeld surface contacts.

**Table 1:** Hydrogen-bond geometry (Å, °)

*D*—H⋯*A*	*D*—H	H⋯*A*	*D*⋯*A*	*D*—H⋯*A*
N1—H4⋯O1^i^	0.91 (2)	1.96 (2)	2.8487 (16)	164.7 (18)
O2—H5⋯N2^ii^	0.99 (3)	2.69 (2)	3.2989 (16)	120.2 (18)
O2—H5⋯O1^ii^	0.99 (3)	1.77 (3)	2.7280 (15)	163 (2)

Symmetry codes: (i) 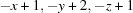; (ii) 
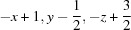
.

**Table 2:** Experimental details

Crystal data	
Chemical formula	C_8_H_5_FN_2_O_2_
*M*_r_	180.14
Crystal system, space group	Monoclinic, *P*2_1_/*c*
Temperature (K)	200
*a*, *b*, *c* (Å)	7.3036 (10), 7.2045 (10), 14.009 (2)
β (°)	94.736 (4)
*V* (Å^3^)	734.61 (18)
*Z*	4
Radiation type	Mo *K*α
μ (mm^−1^)	0.14
Crystal size (mm)	0.34 × 0.32 × 0.06

Data collection	
Diffractometer	Bruker APEXII CCD area detector
Absorption correction	Multi-scan (*SADABS*; Krause *et al.*, 2015 ▸)
*T*_min_, *T*_max_	0.663, 0.746
No. of measured, independent and observed [*I* > 2σ(*I*)] reflections	8386, 2142, 1687
*R*_int_	0.021
(sin θ/λ)_max_ (Å^−1^)	0.705

Refinement	
*R*[*F* ^2^ > 2σ(*F* ^2^)], *wR*(*F* ^2^), *S*	0.040, 0.108, 1.05
No. of reflections	2142
No. of parameters	126
H-atom treatment	H atoms treated by a mixture of independent and constrained refinement
Δρ_max_, Δρ_min_ (e Å^−3^)	0.30, −0.21

Computer programs: *APEX2* and *SAINT* (Bruker, 2014 ▸), *SHELXT2014/4* (Sheldrick, 2015*a* ▸), *SHELXL2016/6* (Sheldrick, 2015*b* ▸), *WinGX* (Farrugia, 2012 ▸), *DIAMOND* (Brandenburg, 2006 ▸), *GOLD* (Chen *et al.*, 2015 ▸), *MOPAC* (Stewart, 2016 ▸), *CRYSTAL EXPLORER* (Wolff, *et al.*, 2012 ▸), *publCIF* (Westrip, 2010 ▸) and *enCIFer* (Allen *et al.*, 2004 ▸).

## supplementary crystallographic information

### Crystal data

**Table:**

C_8_H_5_FN_2_O_2_	*F*(000) = 368
*M**_r_* = 180.14	*D*_x_ = 1.629 Mg m^−^^3^
Monoclinic, *P*2_1_/*c*	Mo *K*α radiation, λ = 0.71073 Å
*a* = 7.3036 (10) Å	Cell parameters from 3387 reflections
*b* = 7.2045 (10) Å	θ = 2.8–30.0°
*c* = 14.009 (2) Å	µ = 0.14 mm^−^^1^
β = 94.736 (4)°	*T* = 200 K
*V* = 734.61 (18) Å^3^	Plate, yellow
*Z* = 4	0.34 × 0.32 × 0.06 mm

### Data collection

**Table:**

Bruker APEXII CCD area detector diffractometer	1687 reflections with *I* > 2σ(*I*)
Radiation source: fine-focus sealed X-ray tube	*R*_int_ = 0.021
φ and ω scans	θ_max_ = 30.1°, θ_min_ = 2.8°
Absorption correction: multi-scan (SADABS; Krause *et al.*, 2015)	*h* = −10→10
*T*_min_ = 0.663, *T*_max_ = 0.746	*k* = −10→7
8386 measured reflections	*l* = −19→19
2142 independent reflections	

### Refinement

**Table:**

Refinement on *F*^2^	Primary atom site location: structure-invariant direct methods
Least-squares matrix: full	Secondary atom site location: difference Fourier map
*R*[*F*^2^ > 2σ(*F*^2^)] = 0.040	Hydrogen site location: mixed
*wR*(*F*^2^) = 0.108	H atoms treated by a mixture of independent and constrained refinement
*S* = 1.05	*w* = 1/[σ^2^(*F*_o_^2^) + (0.0428*P*)^2^ + 0.3741*P*] where *P* = (*F*_o_^2^ + 2*F*_c_^2^)/3
2142 reflections	(Δ/σ)_max_ < 0.001
126 parameters	Δρ_max_ = 0.30 e Å^−^^3^
0 restraints	Δρ_min_ = −0.21 e Å^−^^3^

### Special details

**Table:**

Geometry. All esds (except the esd in the dihedral angle between two l.s. planes) are estimated using the full covariance matrix. The cell esds are taken into account individually in the estimation of esds in distances, angles and torsion angles; correlations between esds in cell parameters are only used when they are defined by crystal symmetry. An approximate (isotropic) treatment of cell esds is used for estimating esds involving l.s. planes.

### Fractional atomic coordinates and isotropic or equivalent isotropic displacement parameters (Å^2^)

**Table:**

	*x*	*y*	*z*	*U*_iso_*/*U*_eq_	
C1	0.40095 (17)	0.78184 (19)	0.56663 (9)	0.0247 (3)	
C2	0.34449 (16)	0.59266 (19)	0.59782 (9)	0.0225 (3)	
C3	0.25830 (15)	0.49961 (19)	0.51303 (8)	0.0219 (3)	
C4	0.18138 (17)	0.3250 (2)	0.49698 (10)	0.0263 (3)	
H1	0.176175	0.236360	0.546869	0.032*	
C5	0.11244 (18)	0.2879 (2)	0.40349 (10)	0.0297 (3)	
C6	0.11742 (18)	0.4116 (2)	0.32854 (10)	0.0306 (3)	
H2	0.068596	0.377653	0.266022	0.037*	
C7	0.19449 (18)	0.5867 (2)	0.34516 (9)	0.0277 (3)	
H3	0.199749	0.674448	0.294847	0.033*	
C8	0.26306 (16)	0.62779 (19)	0.43768 (9)	0.0227 (3)	
F1	0.03607 (14)	0.11739 (14)	0.38491 (7)	0.0459 (3)	
N1	0.34730 (16)	0.79375 (17)	0.47154 (8)	0.0261 (3)	
N2	0.39084 (15)	0.54652 (17)	0.68513 (8)	0.0262 (3)	
O1	0.48449 (15)	0.90064 (15)	0.61615 (7)	0.0324 (3)	
O2	0.34792 (15)	0.36593 (15)	0.70338 (7)	0.0340 (3)	
H4	0.384 (3)	0.889 (3)	0.4351 (15)	0.046 (5)*	
H5	0.409 (3)	0.352 (3)	0.7684 (18)	0.073 (7)*	

### Atomic displacement parameters (Å^2^)

**Table:**

	*U*^11^	*U*^22^	*U*^33^	*U*^12^	*U*^13^	*U*^23^
C1	0.0267 (6)	0.0261 (7)	0.0210 (6)	0.0027 (5)	0.0005 (4)	−0.0002 (5)
C2	0.0219 (5)	0.0254 (7)	0.0201 (6)	0.0032 (5)	0.0008 (4)	−0.0008 (5)
C3	0.0190 (5)	0.0268 (7)	0.0197 (5)	0.0034 (5)	0.0007 (4)	−0.0020 (5)
C4	0.0241 (6)	0.0297 (7)	0.0249 (6)	0.0013 (5)	0.0013 (4)	−0.0016 (5)
C5	0.0261 (6)	0.0307 (8)	0.0318 (7)	−0.0015 (5)	−0.0006 (5)	−0.0086 (6)
C6	0.0262 (6)	0.0418 (9)	0.0230 (6)	0.0020 (6)	−0.0030 (5)	−0.0077 (6)
C7	0.0248 (6)	0.0375 (8)	0.0202 (6)	0.0031 (5)	−0.0013 (4)	0.0005 (5)
C8	0.0197 (5)	0.0280 (7)	0.0203 (6)	0.0037 (5)	0.0006 (4)	−0.0008 (5)
F1	0.0545 (6)	0.0398 (6)	0.0416 (5)	−0.0143 (4)	−0.0063 (4)	−0.0097 (4)
N1	0.0310 (5)	0.0262 (6)	0.0204 (5)	−0.0006 (5)	−0.0023 (4)	0.0032 (4)
N2	0.0293 (5)	0.0279 (6)	0.0214 (5)	0.0006 (4)	0.0010 (4)	0.0018 (4)
O1	0.0454 (6)	0.0279 (6)	0.0230 (5)	−0.0052 (4)	−0.0025 (4)	−0.0014 (4)
O2	0.0434 (6)	0.0314 (6)	0.0263 (5)	−0.0062 (4)	−0.0029 (4)	0.0069 (4)

### Geometric parameters (Å, º)

**Table:**

C1—O1	1.2313 (16)	C5—C6	1.380 (2)
C1—N1	1.3599 (16)	C6—C7	1.393 (2)
C1—C2	1.4994 (19)	C6—H2	0.9500
C2—N2	1.2857 (16)	C7—C8	1.3825 (18)
C2—C3	1.4605 (17)	C7—H3	0.9500
C3—C4	1.3883 (19)	C8—N1	1.4089 (18)
C3—C8	1.4051 (18)	N1—H4	0.91 (2)
C4—C5	1.3898 (19)	N2—O2	1.3674 (16)
C4—H1	0.9500	O2—H5	0.99 (3)
C5—F1	1.3651 (17)		
			
O1—C1—N1	126.68 (13)	C5—C6—C7	119.57 (12)
O1—C1—C2	127.09 (12)	C5—C6—H2	120.2
N1—C1—C2	106.19 (11)	C7—C6—H2	120.2
N2—C2—C3	135.81 (13)	C8—C7—C6	117.44 (13)
N2—C2—C1	117.07 (12)	C8—C7—H3	121.3
C3—C2—C1	106.90 (10)	C6—C7—H3	121.3
C4—C3—C8	120.57 (12)	C7—C8—C3	122.27 (13)
C4—C3—C2	133.56 (12)	C7—C8—N1	127.72 (13)
C8—C3—C2	105.88 (12)	C3—C8—N1	110.00 (11)
C3—C4—C5	115.94 (13)	C1—N1—C8	111.02 (11)
C3—C4—H1	122.0	C1—N1—H4	121.6 (13)
C5—C4—H1	122.0	C8—N1—H4	126.2 (13)
F1—C5—C6	118.18 (12)	C2—N2—O2	112.17 (11)
F1—C5—C4	117.62 (14)	N2—O2—H5	100.2 (15)
C6—C5—C4	124.20 (14)		
			
O1—C1—C2—N2	−1.5 (2)	C5—C6—C7—C8	−0.05 (19)
N1—C1—C2—N2	176.30 (12)	C6—C7—C8—C3	0.59 (19)
O1—C1—C2—C3	−176.99 (13)	C6—C7—C8—N1	179.71 (12)
N1—C1—C2—C3	0.84 (13)	C4—C3—C8—C7	−0.73 (18)
N2—C2—C3—C4	5.3 (2)	C2—C3—C8—C7	179.29 (11)
C1—C2—C3—C4	179.49 (13)	C4—C3—C8—N1	−179.98 (11)
N2—C2—C3—C8	−174.73 (14)	C2—C3—C8—N1	0.04 (14)
C1—C2—C3—C8	−0.53 (13)	O1—C1—N1—C8	177.00 (13)
C8—C3—C4—C5	0.28 (18)	C2—C1—N1—C8	−0.83 (14)
C2—C3—C4—C5	−179.75 (13)	C7—C8—N1—C1	−178.67 (12)
C3—C4—C5—F1	179.86 (11)	C3—C8—N1—C1	0.53 (15)
C3—C4—C5—C6	0.3 (2)	C3—C2—N2—O2	−0.5 (2)
F1—C5—C6—C7	−179.98 (12)	C1—C2—N2—O2	−174.24 (10)
C4—C5—C6—C7	−0.4 (2)		

### Hydrogen-bond geometry (Å, º)

**Table:**

*D*—H···*A*	*D*—H	H···*A*	*D*···*A*	*D*—H···*A*
N1—H4···O1^i^	0.91 (2)	1.96 (2)	2.8487 (16)	164.7 (18)
O2—H5···N2^ii^	0.99 (3)	2.69 (2)	3.2989 (16)	120.2 (18)
O2—H5···O1^ii^	0.99 (3)	1.77 (3)	2.7280 (15)	163 (2)

Symmetry codes: (i) −*x*+1, −*y*+2, −*z*+1; (ii) −*x*+1, *y*−1/2, −*z*+3/2.
