# Supplementary material for: Crystal structure, Hirshfeld analysis and mol­ecular docking with the vascular endothelial growth factor receptor-2 of (3Z)-5-fluoro-3-(hy­droxy­imino)­indolin-2-one
Source: Acta Crystallogr E Crystallogr Commun. 2017 Jun 7;73(Pt 7):987–92. doi: 10.1107/S2056989017008301 (PMC5499275; doi:10.1107/S2056989017008301)
Supplement: Supplementary file 3 [file e-73-00987-sup3.pdf]

SwissTargetPrediction report:

Reference:
Gfeller D., Michielin O. & Zoete V.
Shaping the interaction landscape of bioactive molecules, *Bioinformatics* (2013) 29:3073-3079.

Query Molecule

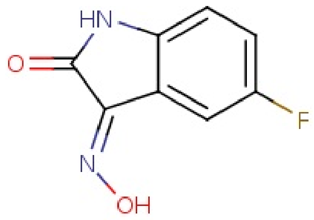

Frequency of Target Class

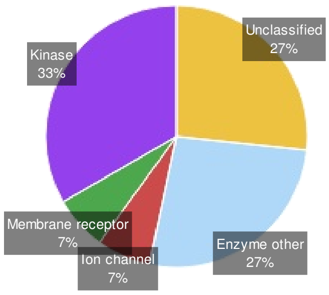

Target	Uniprot ID	Gene code	ChEMBL ID	Probability	# sim. cmpds (3D / 2D)	Target Class
Muscleblind-like protein 1	Q9NR56	MBNL1	CHEMBL1293317		4 / 7	Unclassified
Muscleblind-like protein 2 *(by homology)*	Q5VZF2	MBNL2			4 / 7	Unclassified
Muscleblind-like protein 3 *(by homology)*	Q9NUK0	MBNL3			4 / 7	Unclassified
Microtubule-associated protein tau	P10636	MAPT	CHEMBL1293224		8 / 29	Unclassified
FAD-linked sulfhydryl oxidase ALR	P55789	GFER	CHEMBL1741189		3 / 3	Enzyme
Glutamate receptor ionotropic, NMDA 1	Q05586	GRIN1	CHEMBL2015		40 / 3	Ion channel
Muscarinic acetylcholine receptor M1 *(by homology)*	P11229	CHRM1	CHEMBL216		3 / 10	Membrane receptor
Aldo-keto reductase family 1 member B15 *(by homology)*	C9JRZ8	AKR1B15			1 / 15	Enzyme
Aldo-keto reductase family 1 member B10 *(by homology)*	O60218	AKR1B10	CHEMBL5983		1 / 15	Enzyme
Aldose reductase *(by homology)*	P15121	AKR1B1	CHEMBL1900		1 / 15	Enzyme
Proto-oncogene tyrosine-protein kinase Src	P12931	SRC	CHEMBL267		1 / 1	Tyr Kinase
Tyrosine-protein kinase Fyn *(by homology)*	P06241	FYN	CHEMBL1841		1 / 1	Tyr Kinase
Tyrosine-protein kinase Yes *(by homology)*	P07947	YES1	CHEMBL2073		1 / 1	Tyr Kinase
Tyrosine-protein kinase Fgr *(by homology)*	P09769	FGR	CHEMBL4454		1 / 1	Tyr Kinase
Tyrosine-protein kinase FRK *(by homology)*	P42685	FRK	CHEMBL4223		1 / 1	Tyr Kinase
