# Supplementary material for: Crystal structure, Hirshfeld analysis and mol­ecular docking with the vascular endothelial growth factor receptor-2 of (3Z)-5-fluoro-3-(hy­droxy­imino)­indolin-2-one
Source: Acta Crystallogr E Crystallogr Commun. 2017 Jun 7;73(Pt 7):987–92. doi: 10.1107/S2056989017008301 (PMC5499275; doi:10.1107/S2056989017008301)
Supplement: Supplementary file 4 [file e-73-00987-sup4.pdf]

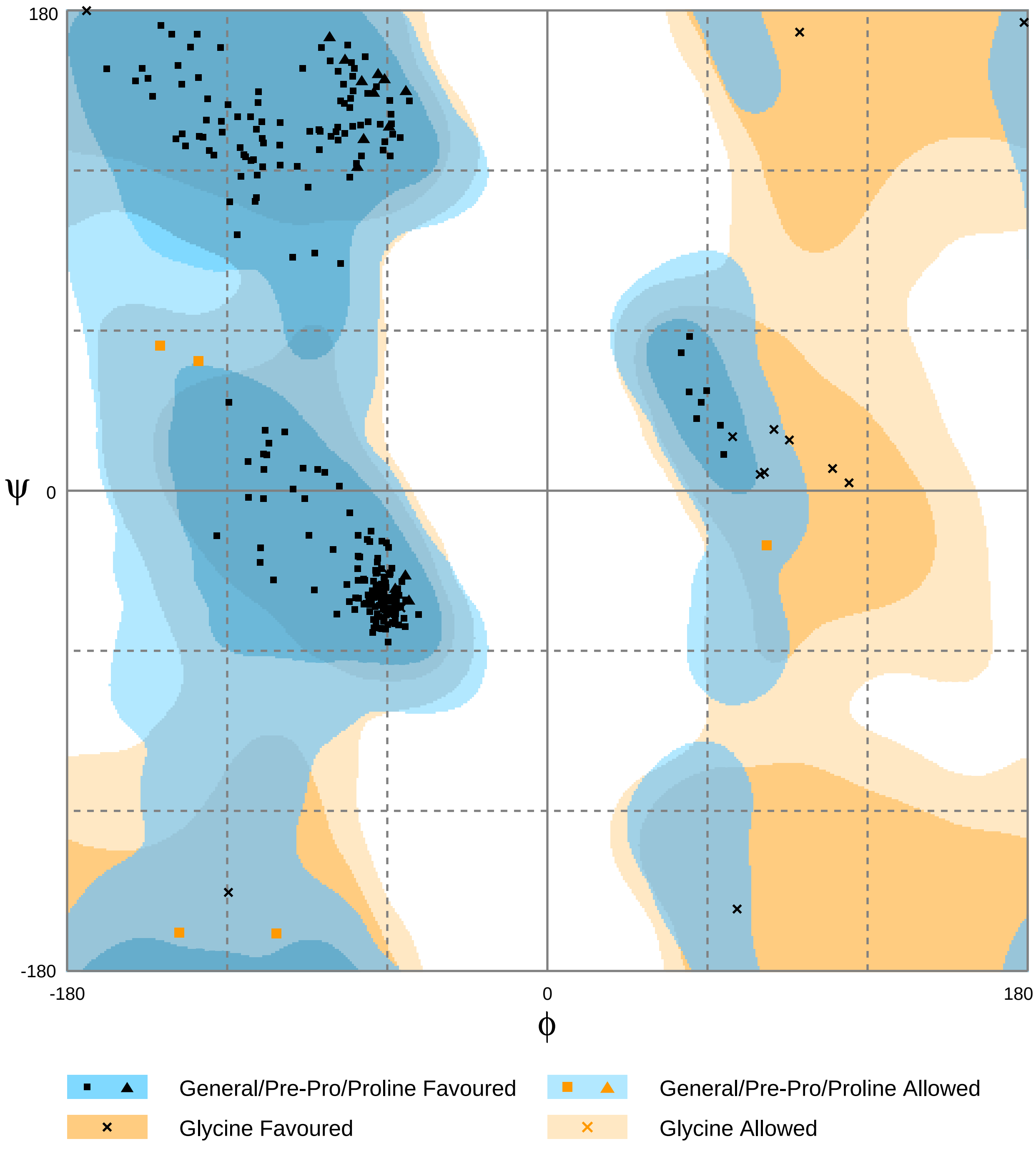

Number of residues in favoured region (~98.0% expected)	:	267 (98.2%)
Number of residues in allowed region (~2.0% expected)	:	5 (1.8%)
Number of residues in outlier region	:	0 (0.0%)

RAMPAGE by Paul de Bakker and Simon Lovell available at http://www-cryst.bioc.cam.ac.uk/rampage/

Please cite: S.C. Lovell, I.W. Davis, W.B. Arendall III, P.I.W. de Bakker, J.M. Word, M.G. Prisant, J.S. Richardson & D.C. Richardson (2002) Structure validation by Cα geometry: φ/ψ and Cβ deviation. *Proteins: Structure, Function & Genetics*. **50**: 437-450

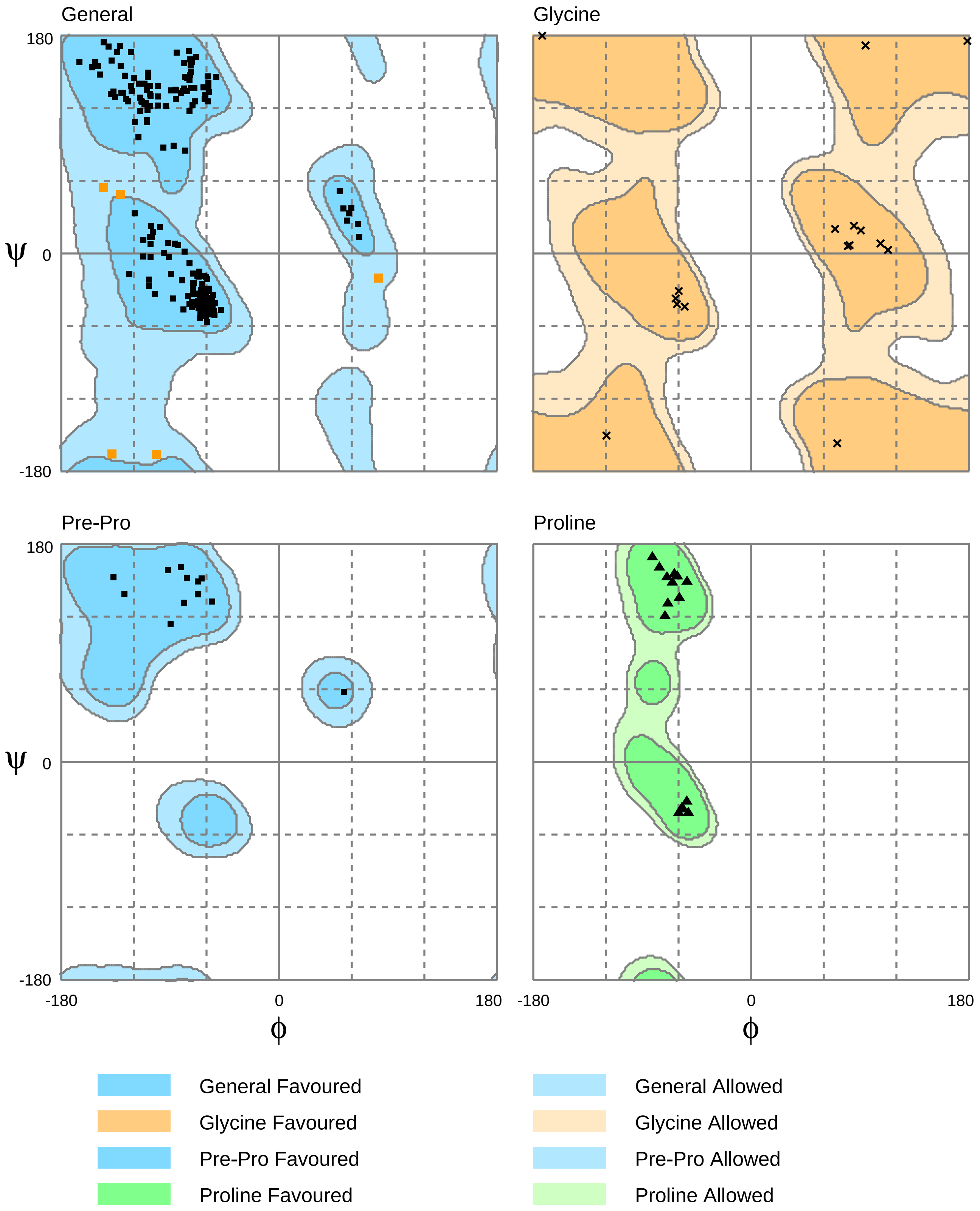

Number of residues in favoured region (~98.0% expected)	:	267 (98.2%)
Number of residues in allowed region (~2.0% expected)	:	5 (1.8%)
Number of residues in outlier region	:	0 (0.0%)

RAMPAGE by Paul de Bakker and Simon Lovell available at http://www-cryst.bioc.cam.ac.uk/rampage/

Please cite: S.C. Lovell, I.W. Davis, W.B. Arendall III, P.I.W. de Bakker, J.M. Word, M.G. Prisant, J.S. Richardson & D.C. Richardson (2002) Structure validation by Cα geometry: ϕ/ψ and Cβ deviation. *Proteins: Structure, Function & Genetics.* **50**: 437-450
