# Supplementary material for: Crystal structure, Hirshfeld analysis and mol­ecular docking with the vascular endothelial growth factor receptor-2 of (3Z)-5-fluoro-3-(hy­droxy­imino)­indolin-2-one
Source: Acta Crystallogr E Crystallogr Commun. 2017 Jun 7;73(Pt 7):987–92. doi: 10.1107/S2056989017008301 (PMC5499275; doi:10.1107/S2056989017008301)
Supplement: Supplementary file 5 [file e-73-00987-sup5.pdf]

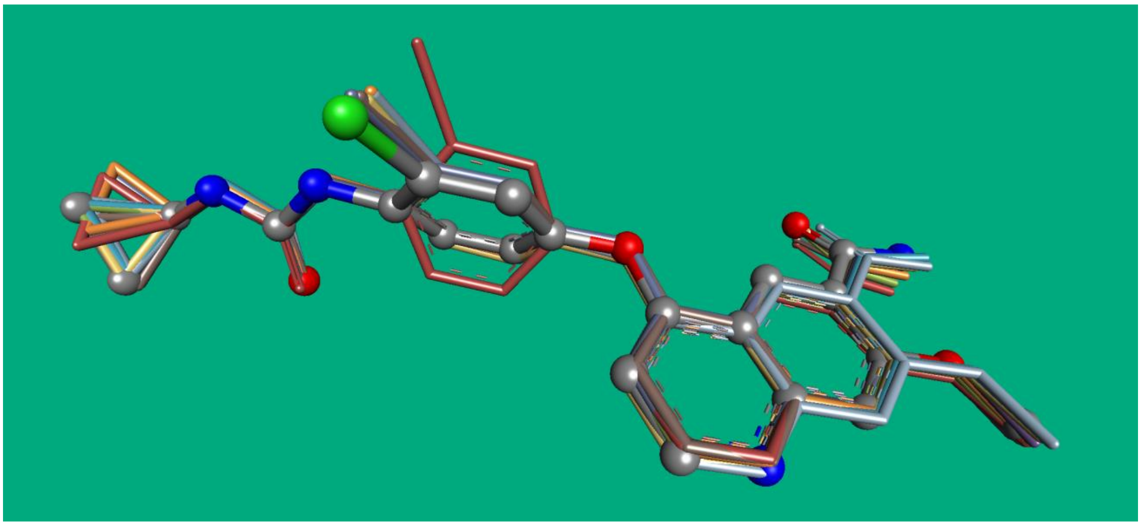

Figure: Re-docking of the Lenvatinib (kinase inhibitor and FDA approved drug) in the binding site of the VEGFR-2 (Okamoto *et al.*, 2015). The higher RMSD amounts to 0.7041 Å. The *C*-, *N*-, *O*-, *Cl*-atoms are grey, blue, red and green balls, respectively.

Okamoto, K., Ikemori-Kawada, M., Jestel, A., von König, K., Funahashi, Y., Matsushima, T., Tsuruoka, A., Inoue, A. & Matsui, J. (2015). *ACS Med. Chem. Lett.* **6**, 89-94.
